# Diagnostic methods for acute otitis media in 1 to 12 year old children: a cross sectional study in primary health care

**DOI:** 10.1186/s12875-019-1018-4

**Published:** 2019-09-11

**Authors:** Pär-Daniel Sundvall, Chrysoula E. Papachristodoulou, Lena Nordeman

**Affiliations:** 1 Region Västra Götaland, Research and Development Primary Health Care, Research and Development Centre Södra Älvsborg, Sven Eriksonsplatsen 4, SE-503 38 Borås, Sweden; 2 Department of Public Health and Community Medicine/Primary Health Care, Institute of Medicine, Sahlgrenska Academy at the University of Gothenburg, Box 454, SE-405 30 Gothenburg, Sweden; 3 Centre for Antibiotic Resistance Research (CARe), Sahlgrenska Academy at the University of Gothenburg, PO Box 480, SE-405 30 Gothenburg, Sweden; 4 Department of Health and Rehabilitation Unit of Physiotherapy, Institute of Neuroscience and Physiology, Sahlgrenska Academy at the University of Gothenburg, Box 455, SE-405 30 Gothenburg, Sweden

**Keywords:** Acute otitis media, Children, Primary health care, Guidelines, Diagnosis

## Abstract

**Background:**

Otoscopy alone has low sensitivity and specificity for acute otitis media (AOM). Otomicroscopy and pneumatic methods are superior to otoscopy. However, these methods require clinical skills. The use of different diagnostic methods for AOM differs between countries and has not been evaluated in Sweden since new guidelines were introduced in 2010. This study aimed to describe the extent of which diagnostic methods and written advice were used for AOM in children 1 to 12 years old.

**Methods:**

In this cross-sectional study all general practitioners (GPs) and specialist trainees in primary care (STs) at 27 primary health care centres in Sweden were asked to complete a self-administrated questionnaire including diagnostic approach and the management of AOM; 75% (111/148) responded to the questionnaire. Outcome Measures: GPs versus STs and their gender, the use of otoscopy, pneumatic otoscopy, otomicroscopy, tympanometry and written advice. Logistic regressions were used to evaluate the association between GPs versus STs and their gender and the use of diagnostic methods and written advice.

**Results:**

To diagnose AOM, 98% of the GPs and STs often or always used otoscopy, in addition to this 17% often or always used otomicroscopy, 18% pneumatic otoscopy and 11% tympanometry. Written advice to parents was provided often or always by 19% of the GPs and STs.

The GPs used otomicroscopy more often than STs, adjusted OR 4.9 (95% CI 1.5–17; *p* = 0.011). For the other diagnostic methods, no differences were found. Female GPs and STs provided written advice more often than male GPs and STs, OR 5.2 (95% CI, 1.6–17; *p* = 0.0061), adjusted for GP versus ST.

**Conclusions:**

Otoscopy was by far the most commonly used method for the diagnosis of AOM. Female GPs and STs provided written advice more frequently than did their male colleagues. GPs used the significantly better method otomicroscopy more often than STs, therefore, it is important to emphasise teaching of practical skills in otomicroscopy in the specialist training programme for general practice. A correct diagnosis is important for avoiding potentially harmful antibiotic treatments, antimicrobial resistance and possible delay of other diagnoses.

**Electronic supplementary material:**

The online version of this article (10.1186/s12875-019-1018-4) contains supplementary material, which is available to authorized users.

## Background

Acute otitis media (AOM) is one of the most common childhood infections, and one of the most frequent reasons for children consulting primary care clinicians and antibiotic consumption in developed countries [1, 2]. The current definition of AOM requires three overall diagnostic criteria: symptoms with rapid onset, findings of tympanic membrane (TM) inflammation, and pus in the middle ear or ear canal [3, 4]. Inspection of the TM is necessary [5]. The same diagnostic methods are used for AOM and secretory otitis media (otitis media with effusion) [2]. Otoscopy alone has low sensitivity and specificity for otitis media (both 61% for middle ear effusion) [6]. Otomicroscopy provides an enlarged view and binocular viewing, which facilitates depth perception and detailed assessment of the TM. Therefore, otomicroscopy is superior to otoscopy (otomicroscopy: sensitivity 87–91% and specificity 89–93% in detecting middle ear effusion) [7, 8] while also providing an opportunity to clean the external acoustic meatus under direct inspection.

Compared to otoscopy, pneumatic otoscopy increases the sensitivity to 94% and specificity to 80%, as pneumatic methods evaluate the degree of mobility of the TM, a reliable sign of the presence or absence of middle-ear effusion, a key criterion for an accurate AOM diagnosis [4, 9, 10]. However, pneumatic methods require clinical skills. Tympanometry is a simple and objective alternative method of assessing TM mobility and middle-ear function with sensitivity similar to pneumatic otoscopy, but lower specificity for AOM, as tympanometry cannot distinguish pus from a non-purulent middle ear effusion [9]. Therefore, inspection of the TM is also necessary. A combination of pneumatic otoscopy and tympanometry further increases diagnostic accuracy and reduces the number of false positive findings of middle ear effusion (sensitivity 93–98%, specificity 93–95%) [2, 4, 11, 12].

The use of diagnostic methods and clinical diagnostic criteria for AOM differs between countries, and are not always clearly described in guidelines [4, 13]. Assessment of AOM can also be affected by several factors, such as knowledge, clinical experience, and difficulties in assessment, such as how to hold a small child, especially if it is crying or avoiding contact [4, 9, 14]. Moreover, cerumen not removed before an examination, poor equipment, or inadequate training in the use of the latter, impair the assessment of AOM [4, 14–17]. Clinicians are somewhat uncertain of the diagnosis of AOM [4, 14, 18–20], and studies often show low adherence to antibiotic guidelines in AOM [4, 13, 21–23]. With a more accurate diagnosis the frequency of false positive AOM is likely to decrease, thus reducing consumption of antibiotics [3]. Further research is needed to investigate the clinician’s diagnostic accuracy and precision in the use of the three AOM criteria [24]. Moreover, the use of different diagnostic methods for AOM in ordinary clinical practice has not been extensively evaluated.

The Swedish guidelines for management of AOM were updated in 2010. Both lower and upper age limits for watchful waiting were lowered, and are now recommended for AOM in children 1 to 12 years without complicating factors or recurrent otitis [4]. The new guidelines emphasize that mobility of the TM should be assessed by pneumatic otoscopy/otomicroscopy and/or tympanometry. Also new in the Swedish guidelines from 2010 is that the AOM diagnosis is considered uncertain when the TM is opaque, discoloured and immobile but not bulging, and watchful waiting is recommended in these uncertain situations as long as there are no complicating factors [4]. The guidelines recommend both oral and written advice on AOM to parents [4], but it is not known to what extent this recommendation has been implemented in Swedish primary health care. Nor is it known if general practitioners (GPs) versus specialist trainees (STs) and their gender are of importance to the extent of which oral and/or written advice is used.

A Swedish survey in 2006 showed that roughly 18% of Swedish GPs used pneumatic otoscopy, 33% otomicroscopy, and 8% tympanometry when diagnosing AOM [2]. It is not known if this changed after the new Swedish guidelines in 2010, nor is it known whether the use of the different diagnostic methods differs between GPs and STs, or if there are any gender differences.

This study aimed to describe the extent of which diagnostic methods were used in the diagnosis of AOM, otoscopy, pneumatic otoscopy, otomicroscopy, tympanometry and the combination of pneumatic otoscopy/otomicroscopy with tympanometry, and investigate how frequently written advice was provided to parents of children aged 1 to 12 years with AOM in primary health care. Furthermore, the aim was to investigate the relationship between GPs versus STs and their gender and the use of diagnostic methods and written advice.

## Methods

### Study design and setting

A cross-sectional study was conducted including GPs and STs from primary health care in Södra Älvsborg County in Southern Sweden, a mixture of urban and rural populations. All GPs and STs (*n* = 154) at all publicly and privately owned primary health care centres (PHCCs) (*n* = 35) in Södra Älvsborg County were invited to participate in the study in 2012. GPs and STs were asked to complete a self-administrated questionnaire including demographic data, diagnostic approach, and the management of AOM (Additional file 1).

### Subjects

Initial contact was made with the managers of the PHCCs (*n* = 35) in Södra Älvsborg. Written information was sent by e-mail, asking for the names of the GPs and STs working at each PHCC. Then, a register with the names of the GPs and STs enrolled in the study was created and coded. An information letter of the study, a questionnaire and a prepaid, addressed envelope was sent by mail to the GPs and STs from whom we obtained contact information. A reminder was sent to those not responding within a month.

To demonstrate whether the use of diagnostic methods differed between GPs and STs we calculated the sample size by assuming that a difference of 20% between STs and GPs was relevant. With a power of 80% and an alpha value of 0.05, 62 participants per group were required.

### Measurements

The self-administrated questionnaire included items for age, gender, GP or ST and years of work experience. Five items evaluated the use of diagnostic methods; otoscopy, pneumatic otoscopy, otomicroscopy, tympanometry, and pneumatic otoscopy/otomicroscopy combined with tympanometry for AOM. Answers ranged from never, seldom, sometimes, often and always. Access to these methods was also queried by a yes or no answer. One item registered change in diagnostic routines of AOM after new guidelines were introduced in 2010. The last item registered whether oral or written advice was provided with watchful waiting. The answers ranged from never, seldom, sometimes, often and always.

### Statistical analysis

Descriptive data were presented using numbers and percentages, median, percentile, mean and standard deviation depending on data level. For comparison between groups the Mann-Whitney test was used for ordinal data, and Student’s t-test for continuous data. The significance level was set at *p* < 0.05.

To evaluate the association between GPs versus STs and their sex for the use of different diagnostic methods, logistic regressions were performed. The frequency of the use of the diagnostic methods was dichotomised into two categories, never using (0) or seldom to always using (1) the method. The use of the different diagnostic methods was used as dependent variables, and GPs versus STs and their sex as independent variables.

Logistic regression was also performed to evaluate the association between GPs versus STs and their sex for the use of written advice. For frequency of the use of oral and/or written advice to parents, the answers were dichotomised into two categories “never to seldom using” (0) or “sometimes to always using” (1) written advice. The use of written advice was used as dependent variable, and GPs versus STs and their sex as independent variables. Analyses were made in SPSS Windows version 21.0.

## Results

**Fig. 1:**
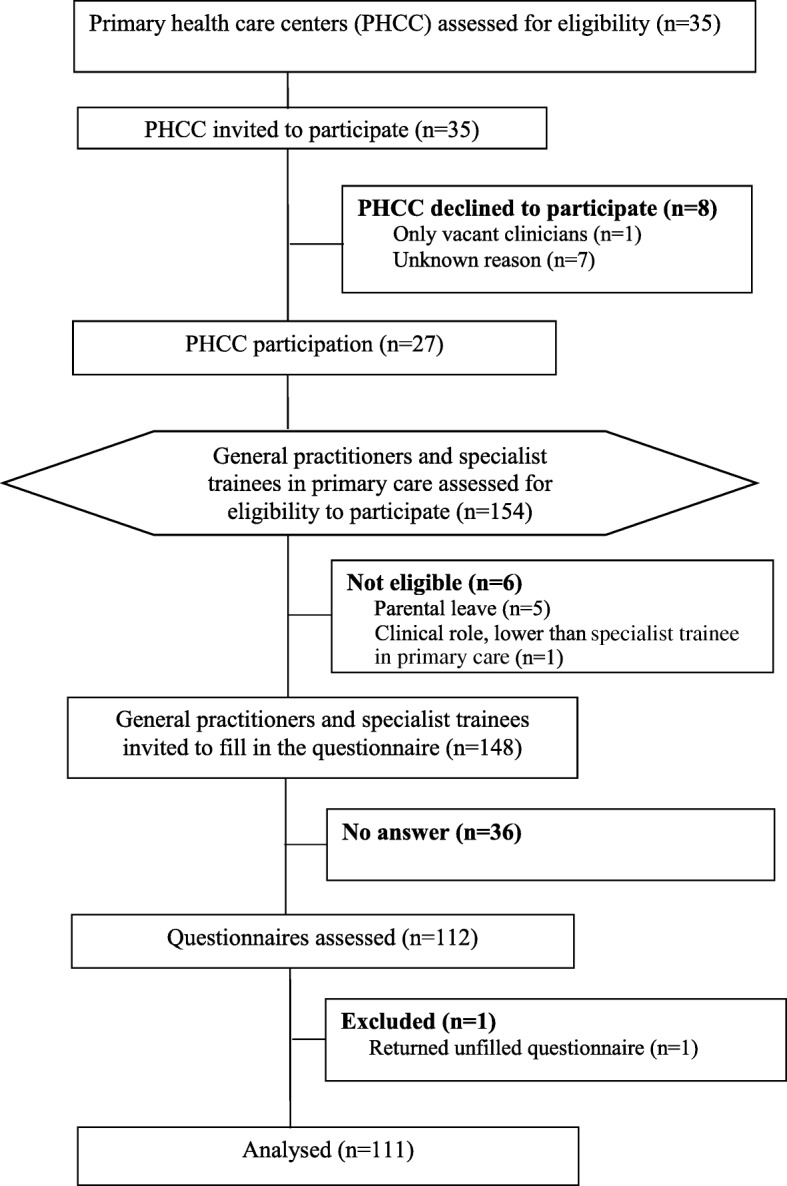
Participants’ flowchart

Among all GPs and STs assessed for eligibility to participate in the study (*n* = 154), six were not eligible: parental leave (*n* = 5) and clinical role lower than ST (n = 1) (Fig. 1. Participants’ flowchart). The remaining 148 GPs and STs were invited to fill in the questionnaire. Seventy-five percent (111/148) of the GPs and STs responded to the questionnaire at 27 of the 35 invited PHCCs (Fig. 1). Group characteristics are presented in Table 1. Sixty percent (64/106) of the GPs and STs reported assessing 5 to 15 children monthly, 1 to 12 years old with suspected AOM (Table 1).

**Table 1:** Demographic data for the study group (*n* = 111)

Gender (female) % (n)	43% (48/111)
Age (years)^1^	47 (11)
Clinical role % (n)	
General practitioner	68% (75/111)
Specialist trainee in primary care	32% (36/111)
Work experience (years)^2^	12 (9.7) -- 10 (3.0–20)
Number of assessed children per month % (n)	
< 5	36% (38/106)
5–15	60% (64/106)
> 15	3.8% (4/106)
Access to all diagnostic methods^3^	81% (90/111)
Not access to % (n):	
otoscopy	0% (0/111)
pneumatic otoscopy	7.2% (8/111)
otomicroscopy	5.4% (6/111)
tympanometry	2.7% (3/111)
pneumatic otoscopy + otomicroscopy	0.0090% (1/111)
otomicroscopy + tympanometry	0.0090% (1/111)
unsure about other methods than otoscopy	1.8% (2/111)

^1^ Mean (SD). Available for analysis (*n* = 110)

^2^ The first figure is the mean value (SD). The second figure is the median (25:th - 75:th percentile)

^3^ Otoscopy, pneumatic otoscopy, otomicroscopy and tympanometry

Sixty-eight percent (51/75) of the GPs were male, while only 33% (12/36) of the STs were male (*p* = 0.00093). When comparing GPs and STs, a statistically significant difference was seen for age (mean (SD)): 53 years (8.1) and 35 years (4.6) respectively (*p* = 0.00032), which was expected, since the STs were being trained as GPs. There was no significant difference between GPs and STs regarding the number of children aged 1 to 12 years assessed monthly with suspected AOM (*p* = 0.29). Female GPs and STs were younger than male (mean (SD)), 43 years (9.5) and 50 years (11), respectively (*p* = 0.00015). Male GPs and STs had nearly twice the professional experience compared to females (mean (SD)), 15 years (7.8) versus 7.6 years (9.9), (*p* = 0.000045). Female GPs and STs saw as many children per month as male GPs and STs (*p* = 0.75).

### The use of diagnostic methods and advice to parents

The use and frequency of diagnostic methods are presented in Table 2. Ninety-six percent (107/111) of the GPs and STs stated always using otoscopy for evaluation of the TM and the use of other diagnostic methods varied (Table 2).

**Table 2:** Use of diagnostic methods in acute otitis media and advice to parents by GPs^1^ and STs^2^

	Never	Seldom	Sometimes	Often	Always
Otoscopy	0% (0/111)	0.90% (1/111)	0.90% (1/111)	1.8% (2/111)	96% (107/111)
Pneumatic otoscopy	29% (29/101)	29% (29/101)	25% (25/101)	16% (16/101)	2.0% (2/101)
Otomicroscopy	15% (15/103)	20% (21/103)	49% (50/103)	15% (15/103)	1.9% (2/103)
Tympanometry	36% (38/105)	24% (25/105)	29% (30/105)	9.5% (10/105)	1.9% (2/105)
Pneumatic otoscopy and/or tympanometry	14% (15/108)	21% (23/108)	38% (41/108)	23% (25/108)	3.7% (4/108)
Combination of pneumatic otoscopy/otomicroscopy and tympanometry	50% (49/98)	26% (25/98)	21% (21/98)	3.1% (3/98)	0% (0/98)
Advice to parents^3^					
Oral	0% (0/111)	0% (0/111)	0.90% (1/111)	5.4% (6/111)	94% (104/111)
Written	41% (40/97)	24% (23/97)	16% (16/97)	12% (12/97)	6.2% (6/97)

^1^ General practitioners (*n* = 75)

^2^ Specialist trainees in primary care (*n* = 36)

^3^ Advice includes pain relieving and follow-up if choice for watchful waiting was made

Ninety-four percent (104/111) reported always providing oral advice, while only 6.2% (6/97) always provided written advice when they chose watchful waiting (Table 2).

### Predictors for the use of diagnostic methods

The GPs used otomicroscopy to a greater extent than STs, adjusted OR 4.9 (95% CI 1.5–17; *p* = 0.011) (Table 3). There was no difference in the use of pneumatic otoscopy, unadjusted OR 2.4 (95% CI 0.99–5.9; *p* = 0.052). When adjusting for sex the OR was 2.1 (95% CI 0.83–5.4; *p* = 0.12) (Table 3). For the other diagnostic methods no differences were seen (Table 3).

**Table 3:** Logistic regression to evaluate the use of diagnostic methods for acute otitis media in children

	Unadjusted odds ratio (95% CI; *p*-value)	Adjusted odds ratio^1^ (95% CI; p-value)
Pneumatic otoscopy		
GP (ST is reference)	2.4 (0.99–5.9; p = 0.052)	2.1 (0.83–5.4; p = 0.12)
Female gender	0.52 (0.22–1.2; *p* = 0.14)	0.64 (0.26–1.6; *p* = 0.34)
Otomicroscopy		
GP (ST is reference)	4.0 (1.3–12; ***p*** **= 0.017**)	4.9 (1.5–17; **p = 0.011**)
Female gender	1.1 (0.36–3.3; *p* = 0.88)	1.9 (0.54–6.4; *p* = 0.33)
Tympanometry		
GP (ST is reference)	0.83 (0.36–1.9; *p* = 0.66)	0.90 (0.37–2.2; *p* = 0.82)
Female gender	1.3 (0.59–3.0; *p* = 0.50)	1.3 (0.54–3.0; *p* = 0.58)
Combination^2^		
GP (ST is reference)	1.1 (0.47–2.5; *p* = 0.83)	1.3 (0.54–3.2; *p* = 0.54)
Female gender	1.7 (0.74–3.7; *p* = 0.22)	1.8 (0.77–4.2; *p* = 0.18)

^1^ The use of diagnostic methods in 1 to 12 year old children consulting primary health care was dichotomised in two categories, never using (0) or seldom to always using (1) the method, adjusted for general practitioners (GPs) versus specialist trainees in primary care (STs) and their sex

^2^ Combination of pneumatic otoscopy/otomicroscopy and tympanometry

Statistically significant findings are bold

### Predictors for the use of advice to parents

Female GPs and STs provided written advice more often than male GPs and STs, OR 5.2 (95% CI, 1.6–17; *p* = 0.0061), adjusted for GP versus ST.

### Changed routines after new guidelines

Thirty-two percent (36/111) of the GPs and STs stated changing diagnostic routines after introduction of the new guidelines, and four reported not knowing whether they had changed. Of those 36 who claimed adhering to the new guidelines, 19 tried watchful waiting and less prescriptions of antibiotics, 11 increased their use of different diagnostic methods, and 6 did not state how they had changed routines.

## Discussion

Otoscopy was by far the most commonly used method for the diagnosis of AOM. Other diagnostic methods such as pneumatic otoscopy, otomicroscopy and tympanometry were used to a lesser extent. The GPs used otomicroscopy more often than STs and female GPs and STs provided written advice to parents more frequently than their male colleagues.

### Strengths and weaknesses of the study

All GPs and STs at all publicly and privately owned PHCCs in Södra Älvsborg County were invited to participate, and 27 of 35 of those invited PHCCs participated. The response rate (74%) at the participating PHCCs was higher than in a previous Swedish survey [2].

The studied county Södra Älvsborg is part of Region Västra Götaland, the second largest region in Sweden. In 2012 there were 1.6 million inhabitants in Region Västra Götaland [25]. Among 201 PHCCs in Region Västra Götaland 43% were privately owned and 57% publicly owned [26]. There were 35 PHCCs, 289,710 inhabitants and 10 municipalities in 2012 in the studied county Södra Älvsborg [27]. Södra Älvsborg consists of a larger town (around 100,000 inhabitants), smaller towns and countryside. Altogether it is reasonable to assume that the studied county Södra Älvsborg is representative for Sweden.

### The studied population

GPs were more often men (68%) compared to STs (33%) (*p* = 0.00093). This gender difference may be explained by more women currently choosing general practice as a specialty [28, 29]. Male GPs and STs were older and had nearly twice the professional experience compared to female, which is assumed to have the same explanation as the gender distribution between GPs and STs.

### Use of diagnostic methods

Otoscopy was by far the most commonly used diagnostic method. Mobility assessment of the TM with pneumatic otoscopy/otomicroscopy or tympanometry was used to a lesser extent when diagnosing AOM. This may be explained by the fact that these methods require technical skills and access to these methods. The results raise questions about the GPs and STs familiarity with the use of various diagnostic methods. STs used otomicroscopy to a lesser extent then GPs, therefore, it is important to emphasise the teaching of practical skills in otomicroscopy in the specialist training programme for general practice. Another study showed that pneumatic otoscopy was minimally used and taught in a family medicine residency program [30]. An Australian study also found low rates of tympanometry (13%) and pneumatic otoscopy (9%) usage among GPs in Australia [31]. Inaccurate diagnosis of AOM might lead to both over- and under diagnosis, resulting in inappropriate antibiotic prescription for AOM with antimicrobial resistance and increased costs to society as a consequence [1, 2, 19]. The results of this study may contribute to the development of specialist training programmes in general practice and antimicrobial stewardship regarding the use of better diagnostic methods for AOM. Correct diagnosis is important for avoiding potentially harmful antibiotic treatments, antimicrobial resistance and possible delay of other diagnoses.

The questionnaire included items with fixed answers partly resembling a previous Swedish survey in 2006 describing the diagnostic routines of AOM [2]. In our study, performed after the introduction of new guidelines for AOM in Sweden in 2010, the use of diagnostic methods other than otoscopy was higher compared to the previous Swedish survey [2]. However, it is difficult to compare these studies as various study designs were used.

To evaluate the association between the GPs versus STs and their sex for the use of different diagnostic methods, logistic regressions were performed. Therefore, we had to dichotomise the frequency of using different diagnostic methods into two categories. The cut-off point never using (0) or seldom to always using (1) the method was chosen on the basis of showing if the GPs and STs consistently abstained from a diagnostic method or used it when it might work in a clinical context. Moreover, to analyse if GP versus ST could predict the use of various diagnostic methods, the cut-off point was considered relevant.

### Predictors for the use of written advice to parents

The Swedish guidelines for AOM recommend the use of written advice [4]. Female GPs and STs provided written advice more often than male GPs and STs in this study. The gender perspective has been shown to play a central role in doctor-patient communication [32, 33].

To be able to evaluate the association between the GPs and STs and their sex for the use of written advice, logistic regressions were performed. As for the diagnostic methods we had to dichotomise the frequency of the use of written advice into two categories. The cut-off point for never to seldom using (0) or sometimes to always using (1) written advice was chosen since it is not relevant to provide written advice at every visit, for example consultations for recurrent AOM where written advice was presented at the previous visit.

## Conclusions

This study showed that 96% of the GPs and STs always used otoscopy for the diagnosis of AOM. Other diagnostic methods such as pneumatic otoscopy, otomicroscopy and tympanometry were used to a lesser extent. GPs used otomicroscopy more often than STs, therefore, it is important to emphasise teaching of practical skills in otomicroscopy in the specialist training programme for general practice. Female GPs and STs provided written advice to parents more frequently than did their male colleagues.

## Additional file

Additional file 1: Self-administrated questionnaire. (DOC 51 kb)

## Data Availability

The data used and supporting the results reported is available on request from the corresponding author.

## Abbreviations

AOM: Acute otitis media
GPs: General practitioners
PHCCs: primary health care centres
STs: Specialist trainees in primary care
TM: Tympanic membrane
