# Supplementary material for: Diagnostic methods for acute otitis media in 1 to 12 year old children: a cross sectional study in primary health care
Source: BMC Fam Pract. 2019 Sep 11;20:127. doi: 10.1186/s12875-019-1018-4 (PMC6739966; doi:10.1186/s12875-019-1018-4)
Supplement: Supplementary file 1 — Self-administrated questionnaire. (DOC 51 kb) [file 12875_2019_1018_MOESM1_ESM.doc]

**Questionnaire regarding diagnostic methods and management
of acute otitis media (AOM) in 1 to 12-year-old children**

***Please answer by circling/ticking the most suitable option for you.***

| 1. Are you a: | General Practitioner | | | | Specialist trainee in primary care | | |
| --- | --- | --- | --- | --- | --- | --- | --- |
| 2. How many years have you worked as a general practitioner/specialist trainee in primary care | _____ years | | | | | | |
| 3. How old are you? | _____ years | | | | | | |
| 4. Are you? | Female | | | | Male | | |
| **5. How many children 1-12 years old with   AOM do you see per month?** | <5 children  5-15 children  >15 children | | | | | | |
| **6. What diagnostic methods do you use to diagnose AOM in children 1-12 years old?** | | | | | | | |
| Otoscopy | Always | | Often | Sometimes | | Seldom | Never |
| Pneumatic otoscopy | Always | | Often | Sometimes | | Seldom | Never |
| Otomicroscopy | Always | | Often | Sometimes | | Seldom | Never |
| Tympanometry | Always | | Often | Sometimes | | Seldom | Never |
| Combination of pneumatic otoscopy/otomicroscopy  and tympanometry | Always | | Often | Sometimes | | Seldom | Never |
| **7. Do you have access to these methods at your primary health care centre?** | | | | | | | |
| | **Yes** |  | | --- | --- | | **No** |  | | **Do not know** |  |   **If you answered no, which methods are missing? _______________________________________________**  **If you answered “do not know”, which methods do you refer to? _________________________________** | | | | | | | |
| **8. In 2010 new guidelines for AOM were introduced in Sweden. Have you, since then,  changed your use of the above diagnostic methods?**   | **Yes** |  | | --- | --- | | **No** |  | | **Do not know** |  |   **If you answered yes, in what way? ______________________________________________________** | | | | | | | |
| **9. Do you advise parents on pain relief and follow-up   when choosing watchful waiting?** | | | Oral advice: | | | | | | --- | --- | --- | --- | --- | | Always | Often | Sometimes | Seldom | Never | | Written advice: | | | | | | Always | Often | Sometimes | Seldom | Never | | | | | | |
